# Eosinophilic Fasciitis: A Case Series

**DOI:** 10.7759/cureus.77615

**Published:** 2025-01-18

**Authors:** Elisa Macedo Brás, António-Pedro Sousa, Nuno Ferreira da Silva, Fernando Salvador

**Affiliations:** 1 Internal Medicine, Centro Hospitalar de Trás-os-Montes e Alto Douro, Vila Real, PRT

**Keywords:** eosinophilic fasciitis, eosinophils, fascia, immunosuppression therapy, skin

## Abstract

Eosinophilic fasciitis (EF) is a rare and frequently underdiagnosed condition, with limited representation in the scientific literature. Early and accurate diagnosis is critical to preventing the progression of the disease and the development of irreversible damage.

EF is characterized by thickening and fibrosis of the subcutaneous fascia and skin, typically affecting the extremities, trunk, and neck, with peripheral eosinophilia occasionally observed. While most cases are considered idiopathic, a thorough evaluation is essential to rule out differential diagnoses or associated underlying conditions. The disease significantly impacts patients’ daily activities and is associated with substantial morbidity.

Management of EF primarily involves the use of corticosteroids and other immunosuppressive therapies. The majority of patients demonstrate favorable responses to treatment, particularly when intervention occurs before the onset of advanced fibrosis.

In this paper, we present a case series involving four patients diagnosed and treated at our institution. We provide a detailed analysis of the diagnostic process, therapeutic approaches, and follow-up strategies employed, contributing to the broader understanding of this rare disease.

## Introduction

Eosinophilic fasciitis (EF) is a rare connective tissue disorder with poorly documented prevalence and gender distribution. It is characterized by thickening, edema, and fibrosis of the subcutaneous fascia and underlying skin, often accompanied by eosinophilic infiltration and peripheral blood eosinophilia. EF shares several clinical features with other autoimmune connective tissue disorders, particularly scleroderma, which constitutes its primary differential diagnosis.

Although the precise pathophysiology of EF remains unclear, it is widely accepted that the condition arises from an inflammatory process originating in the fascia. This inflammation progresses to fibrosis, sclerosis, and, ultimately, irreversible tissue damage [1]. EF has been associated with various potential triggers, including vigorous physical activity, autoimmune diseases (e.g., systemic lupus erythematosus, Sjögren syndrome, primary biliary cirrhosis, and thyroid disorders), graft-versus-host disease, Borrelia burgdorferi infection, hematologic malignancies, and exposure to certain medications, such as statins and immune checkpoint inhibitors [2]. However, the majority of EF cases are considered idiopathic [3].

The clinical presentation of EF primarily involves subacute cutaneous changes in the distal limbs. The condition typically begins with symmetrical erythema and edema accompanied by pain, which later progresses to induration and the formation of skin folds. This evolution culminates in the characteristic peau d'orange appearance of the skin. A hallmark clinical feature of EF is the Groove sign, characterized by linear depressions overlying superficial veins. These depressions become more prominent with limb elevation and the consequent decrease in peripheral venous pressure. This phenomenon is attributed to the restricted mobility of connective tissue surrounding the veins [4]. Involvement of the muscular fascia, with potential extension into the perimysium, may lead to muscle cramps and a mild elevation in muscle enzyme levels. Notably, systemic involvement and Raynaud's phenomenon are uncommon in EF. Their presence should prompt further investigation for alternative or concomitant diagnoses [5].

The diagnosis of EF is established based on the presence of symmetrical induration affecting the limbs, supported by histological evidence of subcutaneous connective tissue fibrosis with fascia thickening and infiltration by eosinophils and monocytes. Alternatively, diagnostic confirmation can be achieved through magnetic resonance imaging (MRI) demonstrating fascia thickening. It is important to note that peripheral eosinophilia is not a consistent finding in EF and does not correlate reliably with disease activity. Other laboratory abnormalities such as elevated erythrocyte sedimentation rate (ESR), C-reactive protein (CRP), and polyclonal hypergammaglobulinemia may also be present [6,7].

The differential diagnosis of EF includes a variety of conditions that may present with overlapping clinical features. These include localized scleroderma, scleromyxedema, nephrogenic systemic fibrosis (in patients undergoing dialysis), dermatomyositis, T-cell lymphoma, and graft-versus-host disease. The presence of systemic involvement should prompt consideration of other conditions, such as hypereosinophilic syndromes, systemic sclerosis, or Churg-Strauss vasculitis. Additionally, during the acute phase of EF, it is essential to rule out alternative diagnoses such as skin infections or stasis dermatitis [8].

The cornerstone of EF treatment is corticosteroid therapy, with prednisolone typically administered at doses of 0.5-1 mg/kg/day [6-9]. In cases of inadequate response or when higher doses of corticosteroids are undesirable, methotrexate at a dosage of 15-20 mg weekly is often the disease-modifying antirheumatic drug (DMARD) of choice [3,9]. Complications of high-dose steroid treatment may include osteoporosis, gastrointestinal bleeding, infections such as tuberculosis reactivation and opportunistic infections, and diabetes, among others. Measures to mitigate these risks may include calcium and vitamin D supplementation, gastroprotective medications like proton pump inhibitors, screening and treatment of latent tuberculosis, prophylaxis for Pneumocystis jirovecii pneumonia, and monitoring and managing blood glucose levels. Other DMARDs, including mycophenolate mofetil [10], hydroxychloroquine, and azathioprine, have also been utilized, though evidence supporting their efficacy is less robust.

There are no clear indications to manage disease recurrence. Potential courses of action include reintroducing or increasing the dosage of corticosteroids, as well as changing or adding another DMARD [11]. For refractory cases, there are documented reports of successful treatment with biologic agents such as rituximab [12] and tocilizumab [13], as well as intravenous immune globulin [14]. Surgical intervention, specifically the release of joint contractures, has also been reported in the literature [15].

Currently, no standardized guidelines exist regarding the optimal corticosteroid tapering strategy or the feasibility of discontinuing treatment following remission. Nevertheless, most patients respond favorably to therapy, with regression of edema and skin thickening, particularly when fibrosis is not yet advanced [11].

## Case presentation

We describe four cases of EF diagnosed and treated at our hospital. The first case involved a 57-year-old woman, with no prior medical records, who presented with complaints of pain, swelling, and thickening of the forearms, hands, legs, and feet. These symptoms had commenced 10 months prior, initially affecting the lower limbs, around the ankles with proximal progression, and subsequently progressing to the upper limbs two months later. The patient reported no inflammatory arthralgia, dysphagia, dyspnea, or Raynaud's phenomenon. She had no family history of connective tissue disorders, nor did she report tobacco, alcohol, or drug use. The patient, a tailor by profession, was unable to continue her work due to the condition.

**Figure 1:**
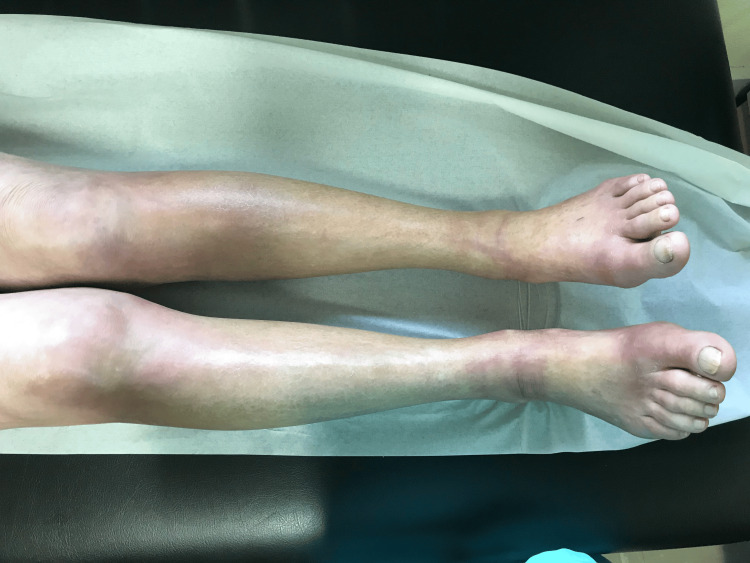
Swelling and thickening observed in both legs

On physical examination, the patient exhibited soft tissue hardening and erythematous skin distal to the elbows and knees (Figure 1).

**Figure 2:**
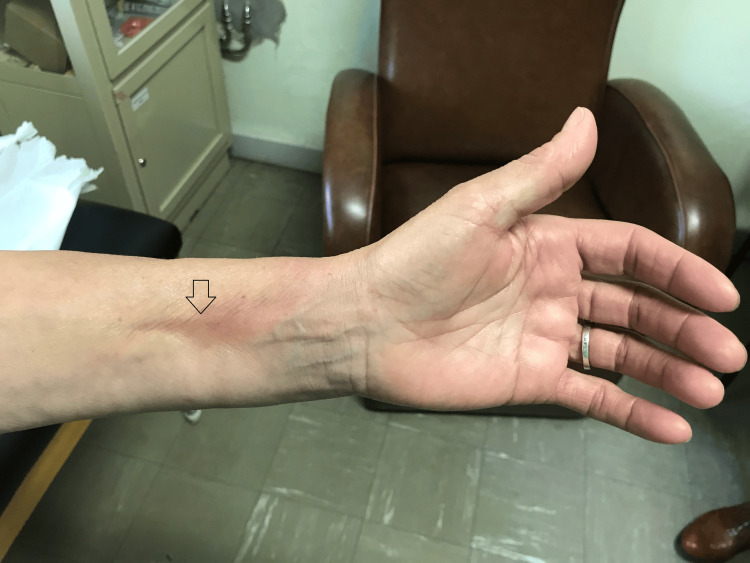
Groove sign in the forearm (arrow)

Linear depressions along the superficial venous pathways in the forearms were observed, consistent with the Groove sign (Figure 2).

The modified Rodnan skin score was 37. No abnormalities were noted in other body segments. Laboratory investigations revealed blood eosinophilia (670 cells/μL; reference range < 300cells/μL), polyclonal hypergammaglobulinemia with immunoglobulin (Ig) G: 1989 mg/dL(reference range: 650-1500 mg/dL); IgA: 458 mg/dL (reference range: 78-312 mg/dL); IgE: 190 UI/ml (reference range < 100 UI/ml), elevated CRP (1.9 mg/dL; reference range <0.5mg/dL), and an ESR of 25 mm/h (reference range < 30mm/h).

Autoantibodies to systemic sclerosis and antinuclear antibodies (ANA’s) were negative, with no complement consumption (C3: 119mg/dL and C4: 19 mg/dL; reference range: C3, 90-180 mg/dL and C4, 12 - 36 mg/dL). Myoglobin and creatine kinase levels were within normal ranges. Nailfold capillaroscopy and endoscopy findings were unremarkable. Electromyography showed no evidence of myositis or polyneuropathy.

**Figure 3:**
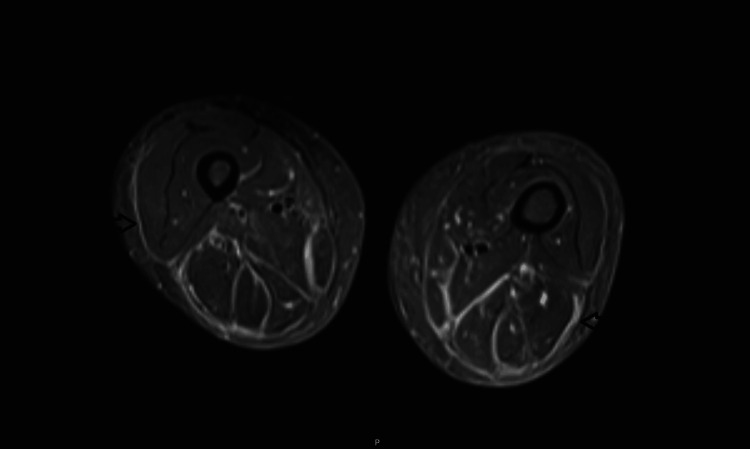
Axial MRI scan of the hips using a STIR sequence, demonstrating fascial inflammation (indicated by arrows) without evidence of muscle involvement MRI: magnetic resonance imaging; STIR: short Tau inversion recovery

**Figure 4:**
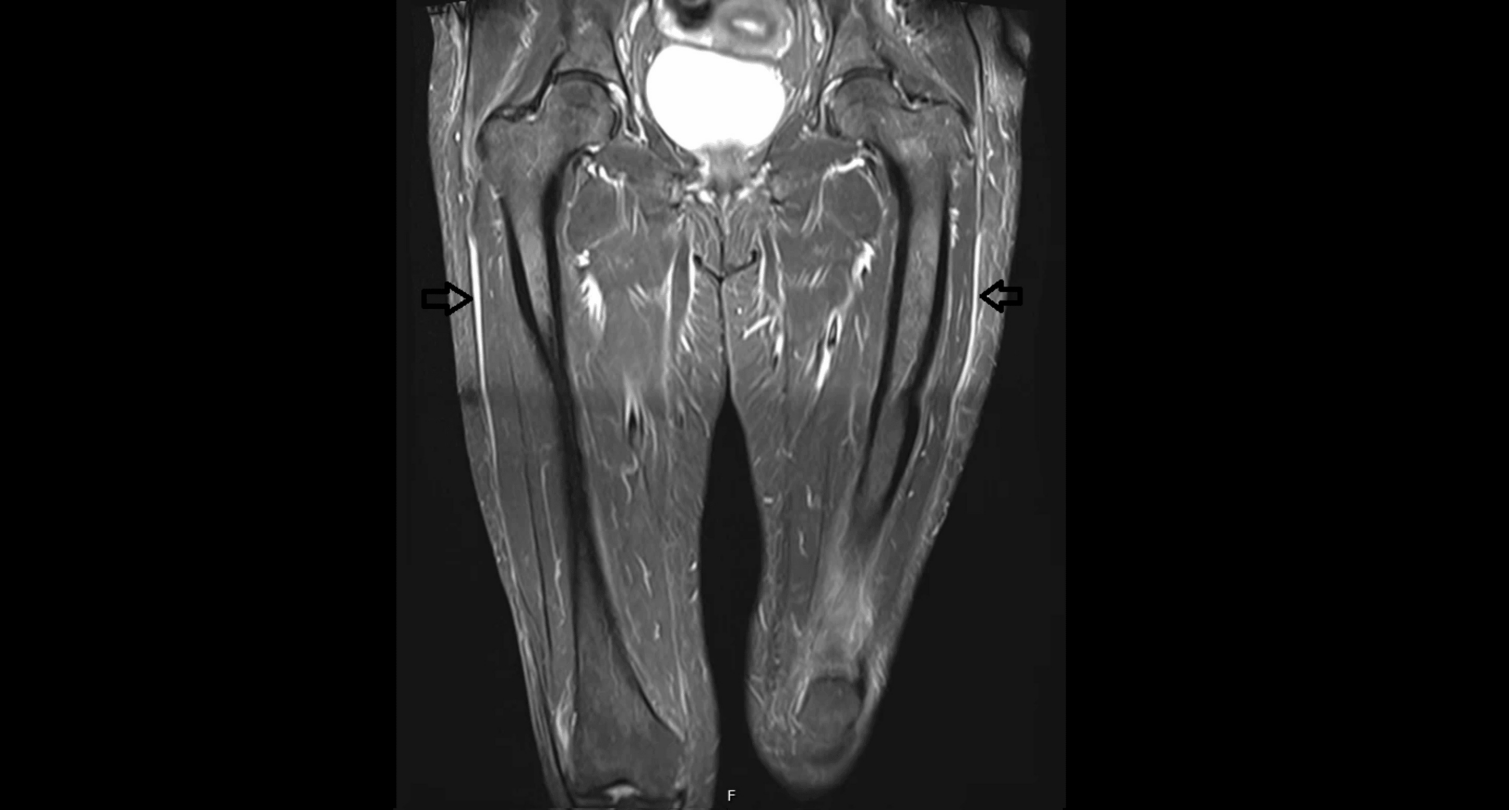
Coronal MRI scan of the hips using a STIR sequence, revealing fascial inflammation MRI: magnetic resonance imaging; STIR: short Tau inversion recovery

Although scleroderma could not be definitively ruled out, histological analysis of the skin revealed dermal and hypodermal fibrosis, loss of adipose tissue, and a minor polymorphic inflammatory infiltrate dominated by lymphohistiocytes with eosinophils. MRI confirmed the diagnosis of EF by demonstrating perifascial edema and effusion bands without muscular involvement (Figure 3 and Figure 4), findings consistent with fasciitis. 

The patient was initially treated with low-dose corticosteroids (20 mg of oral prednisolone daily) in combination with methotrexate (15 mg weekly) because scleroderma could not be definitively ruled out in histology. Baseline liver function tests were within the normal range; pulmonary function tests were not performed due to the absence of respiratory symptoms. After six months, significant improvement in skin hardening was observed in the upper limbs, with slight improvement in the lower limbs. Blood eosinophilia and hypergammaglobulinemia resolved. Prednisolone tapering was initiated, aiming for one year of treatment. At the last visit, 18 months after the diagnosis was made, methotrexate therapy was continued and the patient’s Rodnan score improved to 23.

**Figure 5:**
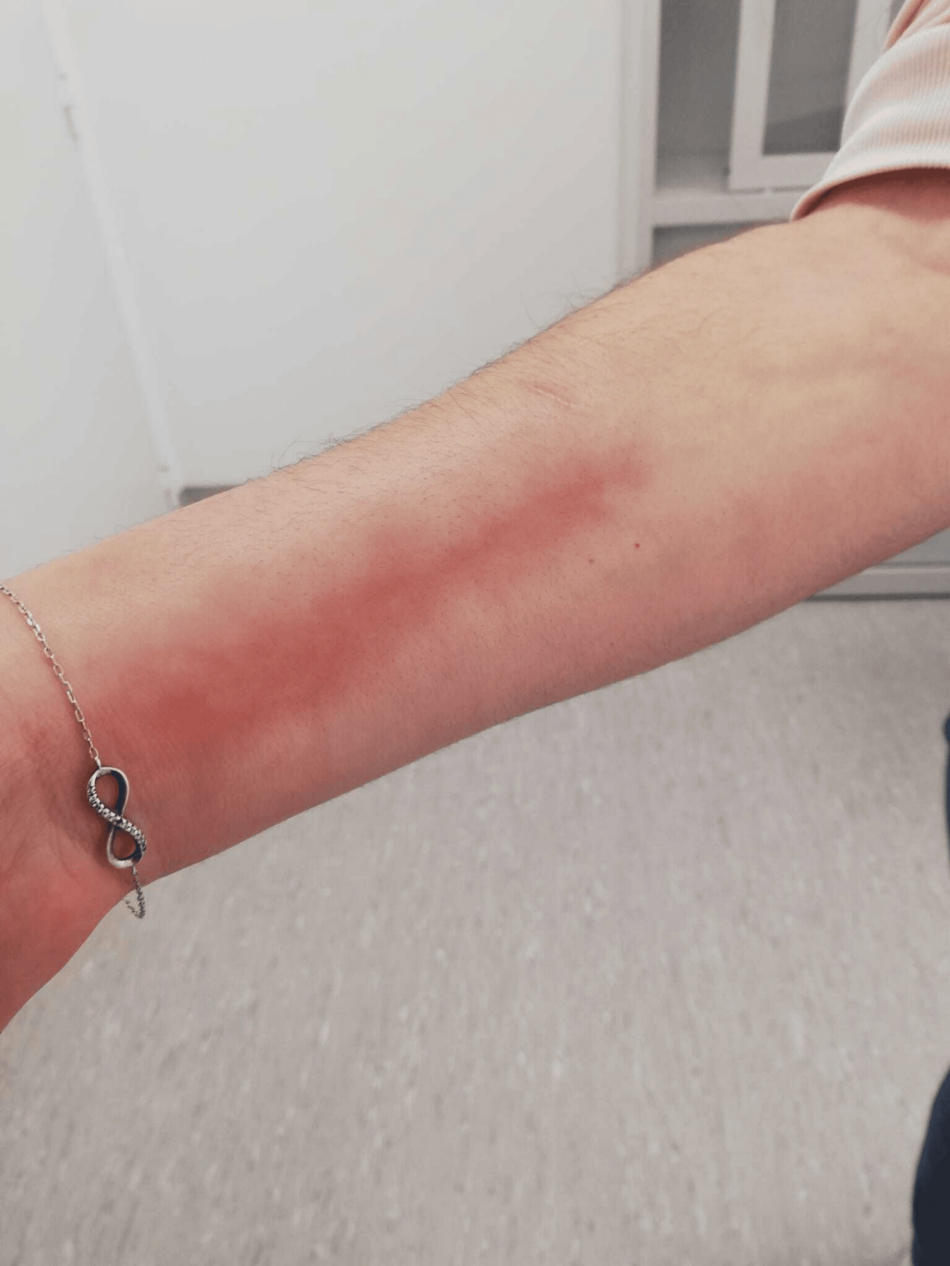
Patient's arm with Groove sign

**Figure 6:**
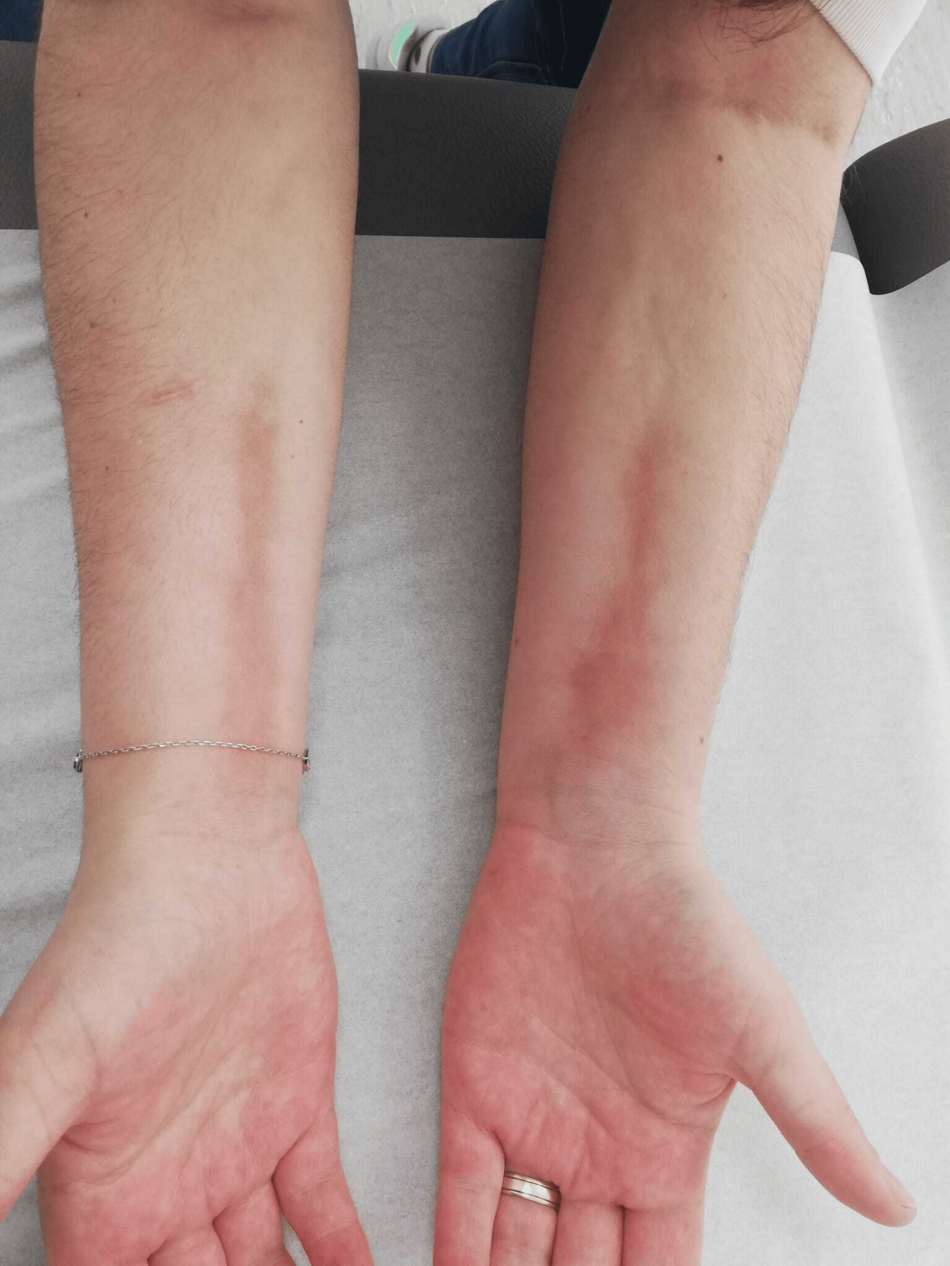
Patient's arms

**Figure 7:**
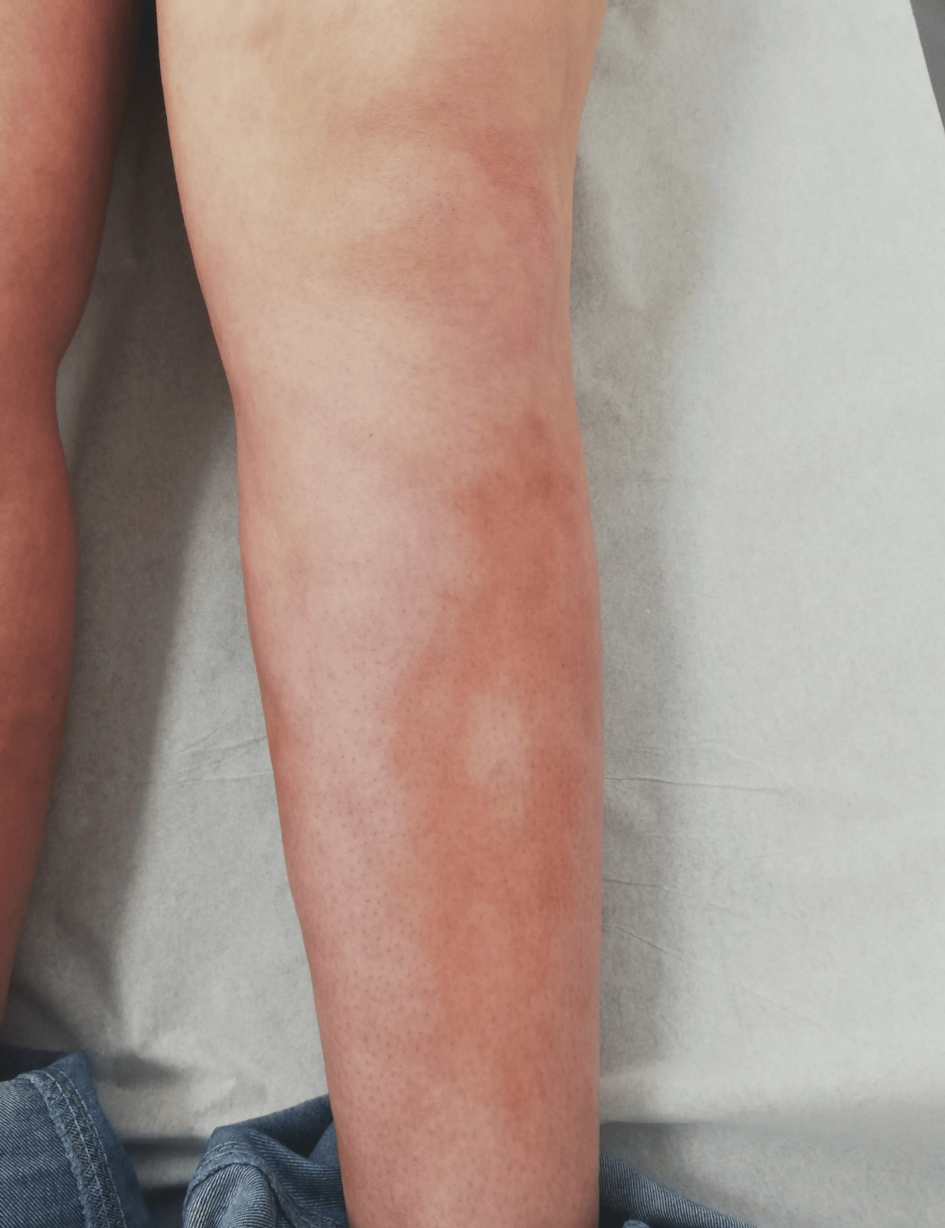
Patient's leg

The second case involved a 26-year-old woman with no significant medical history who presented with a four-month history of skin stiffness, generalized myalgia, and arthralgia of the wrists and ankles. The patient denied fever, weight loss, anorexia, abdominal pain, distal ulcers, Raynaud's phenomenon, or urinary symptoms. On physical examination, there was no evidence of muscle weakness or synovitis; however, skin thickening was observed across the limbs, yielding a modified Rodnan skin score of 48 (Figures 5-7). Laboratory investigations revealed negative antinuclear antibodies, including anti-dsDNA, anti-centromere, and anti-SSA/SSB antibodies. Peripheral eosinophilia (800 cells/µL; reference range < 300cells/μL) was noted, while ESR (16 mm/h; reference range < 30mm/h) and CRP (0.4 mg/dL; reference range <0.5mg/dL) levels were within normal limits; creatine kinase was normal (21 U/L , normal range < 170 U/) and myoglobin was also within the normal range (23 ng/mL, normal range < 58 ng/mL); aldolase was not measured. A computed tomography (CT) scan of the thorax, abdomen, and pelvis showed no significant abnormalities. Histological analysis of a skin biopsy revealed dermal fibrosis with eosinophilic infiltration, confirming the diagnosis of EF. Initial treatment consisted of prednisolone 60 mg/day. The patient tolerated the initial treatment; however due to an unsatisfactory clinical response after eight weeks, methotrexate (15 mg weekly) was added to the regimen. One month after initiating methotrexate, clinical improvement allowed for a gradual tapering of corticosteroids. At the one-year follow-up, the Rodnan score had decreased significantly to 21, with complete resolution of myalgia and arthralgia.

The third patient was a 70-year-old man with a medical history of chronic obstructive pulmonary disease. He had no prior history of allergic reactions, and there had been no recent initiation of new medications or vaccinations. The patient was admitted to the internal medicine ward in May 2017, presenting with a two-month history of asthenia, fever, and bilateral limb edema (both lower and upper limbs). On physical examination, the patient exhibited thickening of the skin, most notably over the forearms, thighs, abdomen, and back, with the forearms being the most affected. Initial laboratory findings showed elevated white blood cell counts, with eosinophilia (maximum of 6920 cells/μL), thrombocytosis (531,000 cells/μL), and elevated CRP (2.28 mg/dL). The ESR was not measured at the time of admission.

The etiological workup revealed negative parasite serologies (Strongyloides, Echinococcus, Fasciola, Toxocara, Leishmania), negative stool parasitology, and negative immune tests for antinuclear antibodies and anti-neutrophil cytoplasmic antibodies. Immunoglobulin levels were within normal limits, and there was no complement depletion. Peripheral blood and myelogram immunophenotyping did not reveal signs of malignancy. Additionally, age-adjusted neoplasia screening did not indicate any malignancies; the etiological workup included endoscopic studies, prostate-specific antigen (PSA) testing, and a thoracoabdominal-pelvic CT scan, all of which did not reveal any malignancies. Electromyography revealed sensorimotor polyneuropathy without myopathic involvement. Initial skin biopsy results were suggestive of dermatomyositis; however, further review of the biopsy slides revealed features consistent with panniculitis and fasciitis, including lymphohistiocytic infiltration in perivascular areas and hypocellular fibrosis. Based on these findings, a diagnosis of EF was established. The patient was started on systemic corticosteroids (prednisolone 60 mg/day). After a brief period of clinical improvement, symptoms recurred upon tapering the corticosteroids. As a result, methotrexate was introduced at an initial dose of 10 mg weekly, which was later increased to 20 mg weekly. This adjustment led to further improvement in symptoms. Corticosteroids were gradually discontinued 18 months after the initiation of methotrexate, which was maintained as the primary therapy. However, four years after the initial diagnosis of EF, the patient was diagnosed with esophageal neoplasia and ultimately succumbed to the progression of the disease.

**Figure 8:**
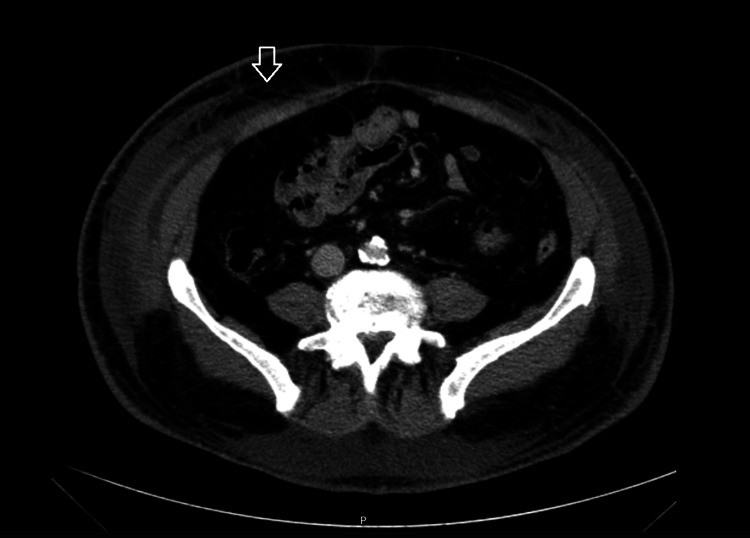
Thoracoabdominal CT scan of patient 4

The last case was a 56-year-old man with a medical history including dyslipidemia, aortic insufficiency, and a smoking history of 33 pack years. The patient was admitted to the internal medicine ward in June 2013, presenting with myalgias and swelling affecting the lower and upper limbs, thorax, and abdomen for a duration of six months. He denied experiencing arthralgia, Raynaud's phenomenon, or respiratory symptoms. Initial investigations revealed blood eosinophilia (1680 cells/μL). Nailfold capillaroscopy demonstrated only minor microvascular changes. Bone biopsy, stool parasitology, and electromyography were all normal. Viral markers showed a pattern consistent with hepatitis B vaccination, and immunoglobulin levels (G, A, and M) were within normal limits, with a slight elevation in immunoglobulin E (246 IU/mL). ANAs were positive at a titer of 1:640, while anti-cyclic citrullinated peptide antibodies and anti-neutrophil cytoplasmic antibodies (ANCAs) were negative. The FIP1 PDGFRA mutation, indicative of systemic mastocytosis, was negative. Upper and lower endoscopy revealed no pathological findings, and prostate-specific antigen levels were normal. A thoracoabdominal CT scan showed increased density and infiltration of subcutaneous fat, with diffuse edema in the soft tissues, most prominent in the pelvic region (Figure 8). A subcutaneous tissue biopsy was performed, which revealed pronounced panniculitis and fasciitis with eosinophilic infiltration. Based on these findings, a diagnosis of EF was made. The patient was initially treated with systemic corticosteroids (prednisolone 60 mg/day), which led to significant symptomatic improvement. However, upon tapering the prednisolone after two months of treatment, the observed improvement in skin thickening ceased, prompting the introduction of methotrexate. Due to minimal clinical improvement, hydroxychloroquine was added to the treatment regimen for a period of four months, but no beneficial effects were noted. After 18 months of treatment, the patient exhibited only minor thickening in the lower limbs, while the upper limbs, thorax, and abdomen had completely improved. The persistent thickening of the lower limbs was considered to represent fibrosis, and prednisolone was discontinued. Methotrexate was also withdrawn three years after treatment initiation, and the patient has not experienced any relapses to date. 

## Discussion

In this paper, we present four cases of a disease first described by Schulman 50 years ago [16].

Since its first description, only a limited number of cases have been reported, leaving a scarcity of information about the condition, including epidemiological data. A retrospective study conducted in a region in the northeast of France demonstrated a prevalence of 14 per million inhabitants in that population [17]. As indicated by retrospective cohorts, the age of diagnosis is typically in the sixth decade of life [9,11,18]. The incidence of the condition between genders was not similar between cohorts, with some favoring women (68%) [9], others with similar incidence (49% of women) [11,18], and a Japanese report with a male: female rate of 2.3:1 [18].

Pathophysiology was not fully understood and may be related to inflammatory processes, as described in the introduction. Most of the patients exhibit a positive response to therapy involving immunosuppressant medications, such as glucocorticoids. On the other hand, signs of proximal extension of the disease or progression to fibrosis, as evidenced by extension to the trunk and peau d'orange, are associated with a more unfavorable prognosis [19].

The clinical presentation is characterized by signs and symptoms that overlap with other autoimmune disorders (such as scleroderma), including skin thickening and joint stiffness, which may contribute to underdiagnosis. Histopathological analysis remains the gold standard for diagnosis, although MRI is also valuable in the diagnostic work-up. In more severe cases, CT scans may reveal fascial inflammation.

Treatment primarily involves immunosuppressive therapy. While the literature advocates for the use of higher doses of corticosteroids, our findings indicate favorable outcomes with lower doses of prednisolone combined with methotrexate, with potential fewer side effects of corticosteroid use. One patient was treated with hydroxychloroquine; however, no clinical benefit was observed. Notably, one patient developed cancer three years after the initial diagnosis, despite negative cancer screening during the disease's early course, leaving the potential association with paraneoplastic syndrome uncertain. The triggers for the disease in the remaining patients were not identified. None of the other patients had recently started a new medication prior to the diagnosis of EF, nor did they exhibit any signs of infection or exposure to potential environmental toxins that could have triggered the disease. One patient was a tailor, suggesting that repetitive trauma might have contributed to the inflammatory response. None of the patients experienced relapse following the initiation of methotrexate therapy.

Clinical outcomes demonstrated regression of edema and skin thickening in most cases, including those with a prolonged disease course. Our patients reported complete remission; we interpreted remission as an improvement in skin condition, characterized by reduced edema and thickening, accompanied by the normalization of eosinophilia, inflammatory markers (ESR and CRP), and hypergammaglobulinemia, accompanied by significant improvements in the quality of life and skin appearance.

## Conclusions

Awareness of this rare condition is crucial, particularly in the differential diagnosis of systemic sclerosis, as the treatment approaches may differ significantly. Accurate diagnosis of EF is essential to prevent irreversible fibrosis, joint contractures, and other complications that can severely compromise patients' quality of life.
